# Tool for Designing Breakthrough Discovery in Materials Science

**DOI:** 10.3390/ma14226946

**Published:** 2021-11-17

**Authors:** Michiko Yoshitake

**Affiliations:** Research Center for Functional Materials, National Institute for Materials Science, Tsukuba 305-40044, Japan; yoshitake.michiko@nims.go.jp; Tel.: +81-298-863-5696

**Keywords:** knowledge database, scientific principles, material property relationship, network-type database, interdisciplinary, multidisciplinary, graph search, wide perspective

## Abstract

A database of material property relationships, which serves as a scientific principles database, and a database search system are proposed and developed. The use of this database can support a broader research perspective, which is increasingly important in the era of automated computer-aided experimentation and machine learning of experimental and calculated data. Examples of the wider use of scientific principles in materials research are presented. The database and its advantages are described. An implementation of the proposed database and search system as a prototype software is reported. The usefulness of the database and search system is demonstrated by an example of a surprising but reasonable discovery.

## 1. Introduction

**Figure 1:**
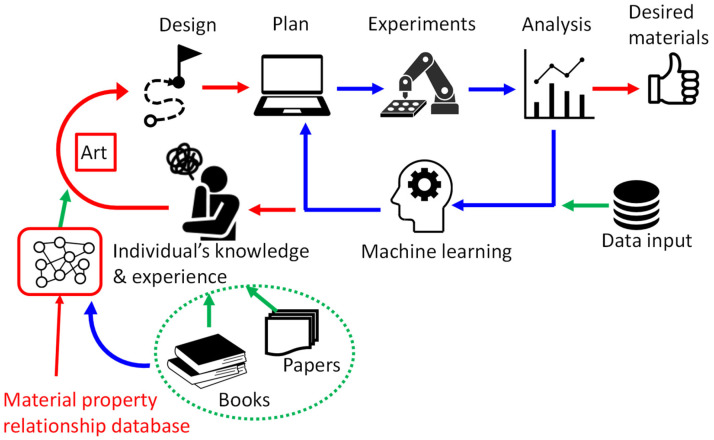
Schematic representation of research process consisting of automated loop with computer aid (**blue**) and human involvement in the process (**red**). Green arrows indicate information inputs.

In conventional materials research and development (R&D), researchers explore materials or synthesis processes based on known materials or processes by modifying one or two conditions in the composition or process (conventional search area). The entire search area is very large, for example, the number of five-element systems composed of any combination of 76 practical elements (excluding inert gases and radioactive elements from the periodic table) can be briefly estimated as follows: the number of permutations of five elements from 76 elements (choosing from the largest content) is 76!/(76−5)!, where ! means factorial. If the compounds containing the same five elements but with different compositions of 1 at% are regarded as different compounds, the total number of possible compounds of the five-element system is approximated by 76!/(76−5)! ∗ (100−4)^5^ ∗ (1/2)^4^, which is larger than 10^17^. Here, (100−4)^5^ (96 at% is the possible maximum concentration) is possible variation of compositions without considering the order in composition and (1/2)^4^ is for taking the order of five elements in consideration. To increase the search speed, high-throughput experimental techniques [1,2,3] and automated experimental systems using robotics techniques [4,5,6] have been developed recently. Machine learning techniques using accumulated data or output data from high-throughput experiments have been introduced in materials R&D [7,8,9,10,11]. Machine learning is a powerful tool for optimizing compositions or process parameters within systems (for example, to find a local minimum) where data are given (that is, the search area consists of various numerical input data). However, because machine learning requires numerical input data, its applications are limited to systems where numerical data for learning exist. By contrast, innovative materials or processes have often been discovered in systems far from existing or explored systems. For example, carbon alloy catalysts for fuel cells [12,13] have no metallic components but contain only carbon and nitrogen, whereas most researchers have tried to decrease the Pt or precious metal content of catalysts. Carbon alloy catalysts could not have been discovered by machine learning using existing data on catalysts containing Pt and/or other metals. To develop these catalysts, it appears that the inventor considered basic scientific principles without being limited by commonly used approaches. The scientific principles and functional mechanism are essentially the same as those of known systems. Here, the knowledge of the inventor appears to have contributed to the discovery. Figure 1 schematically illustrates the automated experiment and machine learning loop (blue lines) and human contribution (red lines) in computer-aided materials R&D. The blue loop in Figure 1 is still under development; however, it is gradually becoming apparent that the red path will become increasingly important in the future. Here, the problem is that individuals acquire knowledge mainly by reading books and papers, which limits the broadness of a field and often results in a narrow outlook on possible approaches. For breakthrough discovery, it is important to support a broader perspective.

**Figure 2:**
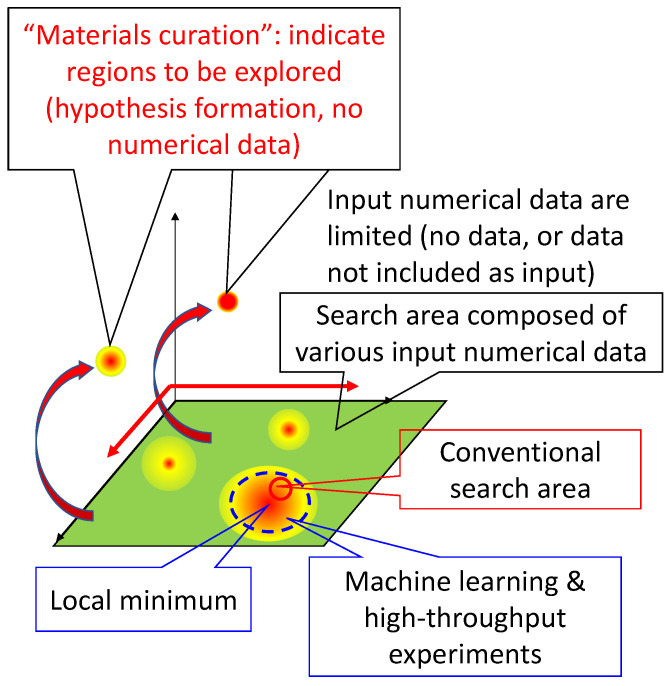
Schematic representation of search space with numerical input data (conventional or machine learning) and without numerical input data (materials curation).

The author has tried to obtain a broader perspective and has made discoveries, which will be described later. On the basis of these experiences, the author proposed “materials curation”, a method of interdisciplinary utilization of scientific principles to solve problems or search for materials from this wider perspective [14,15,16,17,18]. In this method, searches for materials or solutions are conducted beyond the search space in which numerical data are available, as shown schematically in Figure 2, where red indicates more desirable values of target material properties and green indicates less desirable values. To make this method available to many researchers, the author made the concept of a database of scientific principles in materials science [16,17,18]. The database of scientific principles is used in the third and fourth stages of “materials curation”, where the stages are divided into (1) detach from common approaches, (2) consider what the user wants (not needs), (3) describe conditions that satisfy the wants from viewpoint of scientific principles, (4) list methods that can satisfy the conditions in principle, (5) test the method one by one using numerical data, and (6) get new solutions for the wants [16]. On the red path in Figure 1, where the knowledge of an individual human is required, knowledge of scientific principles is acquired mainly from books. The interdisciplinary utilization of scientific principles requires knowledge from multiple fields. However, it is somewhat difficult for individuals to read many books from a broad range of fields. Developing and sharing a database of material property relationships to serve as a database of scientific principles (Figure 1, bottom left) would at least partially solve this problem.

**Figure 3:**
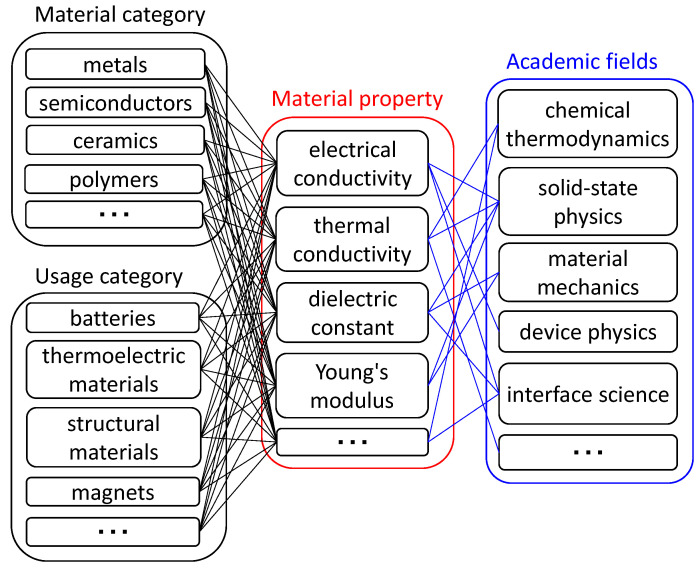
Schematic relationships among material properties (usually categorized by material type or usage; black lines) and scientific principles (usually categorized by academic fields; blue lines).

Interdisciplinary support is realized by associating material properties not with material types or material usage but with academic fields, as shown in Figure 3. For example, the electrical conductivity is determined by the same principle described in solid-state physics regardless of the value. Metals, semiconductors, and ceramics (which are typically insulators) have different conductivity values, but those values are determined mainly by carrier density, which depends primarily on band gap energy. Here, the electrical conductivity, carrier density, and band gap energy (each of which is a material property) are connected through solid-state physics (blue lines in Figure 3). Because associations among material properties are made based on published electronic textbooks, the names of the academic fields are mostly based on titles or categories of textbooks from publishers. This article describes the database of material property relationships and the system for searching these relationships.

## 2. Examples of Knowledge Utilization

Here, examples of knowledge utilization by the author are presented to explain the process of perspective broadening.

### 2.1. Substrate for the Growth of Ultra-Thin Atomically Flat Epitaxial Alumina Film

**Figure 4:**
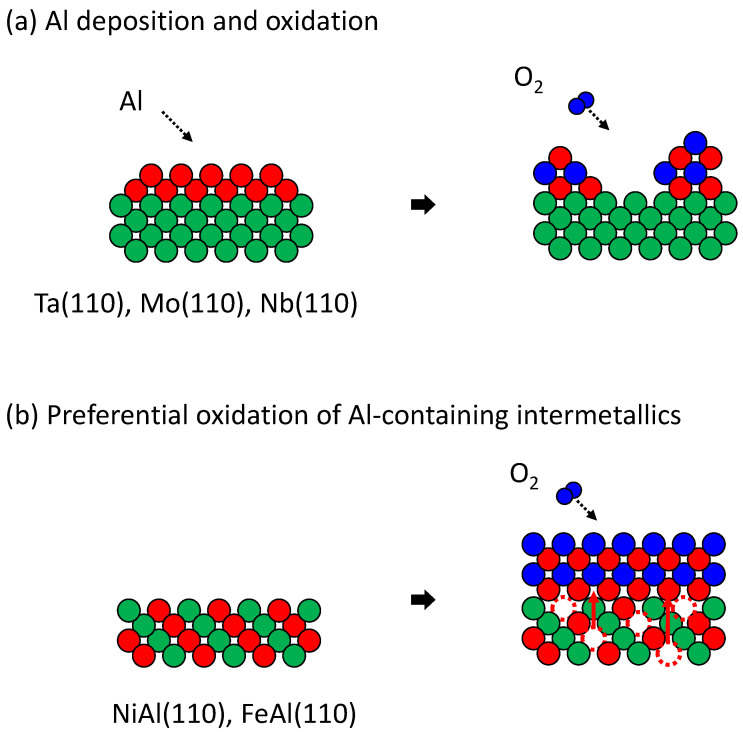
Schematic representation of alumina growth by (**a**) Al deposition and oxidation on high- melting-temperature bcc metal (110) surfaces, and (**b**) preferential oxidation of (110) surface of Al-containing intermetallics having bcc-like structure.

Thin epitaxial alumina films have been grown for the study of electron tunneling, model catalysts and so forth. The most popular substrate used for model catalysts is NiAl(110), where the growth of atomically flat, 0.5 nm thick epitaxial alumina is well known [19]. However, it has been found that a thickness of 0.5 nm is not sufficient to avoid the effects of the metallic underlayer (in this case, NiAl). Therefore, many attempts have been made to use other (metallic) substrates. Figure 4 briefly summarizes the results of these attempts. Two types of substrates have been investigated: the (110) plane of pure body-centered cubic (bcc) metals with high melting temperature such as Ta(110) [20] and Mo(110) [21], and the (110) plane of Al-containing intermetallic compounds such as NiAl(110) and FeAl(110) [22]. On the former type of substrate, aluminum is deposited and then oxidized at high temperatures so that it crystalizes. Alumina is known to grow epitaxially but does not form flat films. The reason is that aluminum–oxygen bonds are so strong that in the first step of oxidation, aluminum atoms agglutinate and become islands. This kind of growth is well known to occur in molecular beam epitaxy (MBE) [23]. For Al-containing intermetallic compounds, preferential oxidation produces flat epitaxial alumina films, but the thickness is less than 1 nm, which is insufficient to avoid the effects of the substrate. In the preferential oxidation of Al-containing intermetallic compounds, O atoms react individually with Al atoms on the upper surface because there is no Al–Al bonding at the surface, and agglutination of Al atoms does not occur. If the Al atomic content is less than stoichiometric, Al atoms below the surface diffuse to the surface and bind with O atoms. Because O atoms do not agglutinate, the diffusion of Al atoms is the rate-determining process. Therefore, the agglutination of Al atoms does not occur, and atomically flat epitaxial films are produced. This mechanism is used in MBE, although the supply of metallic atoms is not controlled by diffusion from a substrate but by beam flux, for example, in the growth of GaAs [23]. Thicker alumina epitaxial layers (slightly thicker than 0.5 nm) can be grown by alternately suppling Al and O under controlled conditions [24]. The thickness is limited to less than 1 nm because of the symmetry mismatch of the crystal planes. In ultra-thin (nanometer-order) epitaxial alumina films, oxygen atoms typically align in sixfold symmetry on the plane parallel to the surface. The crystal structure of NiAl and FeAl is bcc-like, where atoms are aligned quasi-hexagonally but do not have sixfold symmetry on the (110) plane. The symmetry mismatch between the substrate and alumina film causes strain, which is thought to prevent further growth of epitaxial alumina. This hypothesis is supported by the fact that when a thicker layer of alumina was grown on NiAl(110) by further deposition of Al and O, the structure changed at a thickness of 0.84 nm, and the alumina became amorphous when the thickness reached 1.62 nm [24,25].

The above findings suggest the possibility of using Al-containing alloys that have a crystal plane with sixfold symmetry. The author was successful in finding such alloys that fulfill the conditions and demonstrated the growth of 1–4 nm thick atomically flat alumina films using Cu-9Al(111) as a substrate [26,27,28]. The key was to expand the search space beyond intermetallic compounds, which rarely have a plane with sixfold symmetry, and consider alloys as candidate materials.

### 2.2. Thermoelectric Materials

**Figure 5:**
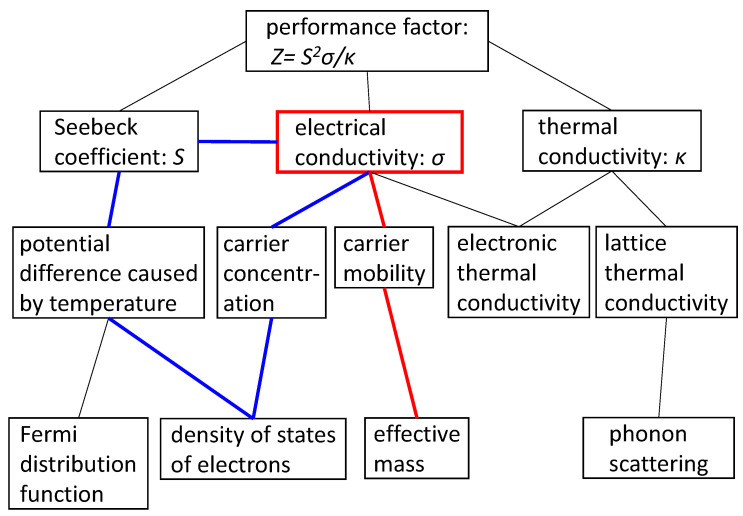
Relationships among various properties affecting the performance factor of thermoelectric materials.

In thermoelectric materials, a voltage is generated between two edges of a material, which are kept at different temperatures. When the two edges are electrically connected via a load, current flows, which can be used as electric power. The efficiency of power generation is expressed as Z = *S^2^σ/κ*, where *S* is the Seebeck coefficient, *σ* is the electrical conductivity, and *κ* is the thermal conductivity. In the early stage of intense research on thermoelectric materials around the beginning of the 2010s, the Seebeck coefficient and electrical conductivity were thought to have a trade-off relationship, and therefore most research focused on controlling the thermal conductivity by fabricating nano structures. However, the author demonstrated that the trade-off can be partially avoided [29,30]. By considering the scientific principles of voltage generation by placing samples of the same material at different temperatures in contact (temperature difference causes difference in electron distribution, accordingly the Fermi level difference, but the shape of density of states (DOS) is the same), and of voltage decrease due to current flow, we can draw a diagram of the relationship between *S, σ, κ*, and the quantities that determine *S, σ*, and *κ*, as shown in Figure 5. [31,32]. One reason for the trade-off relationship is doping, which does not change the main DOS but increases the impurity states (and thus increases *σ*); consequently, the Fermi level changes, decreasing the generated voltage thus *S*. However, this explanation between *S* and *σ* applies only for doping. A comparison of materials with differently shaped DOSs reveals that there is no trade-off relationship [31]. The reason is that the shape of the DOS depends on the carrier mobility, which is determined by the effective mass of electrons. Therefore, a search for materials considering not the DOS but the shape of the DOS would identify materials that have both large Seebeck coefficients and high electrical conductivity.

### 2.3. Prediction of Work Function from Vickers Hardness

**Figure 6:**
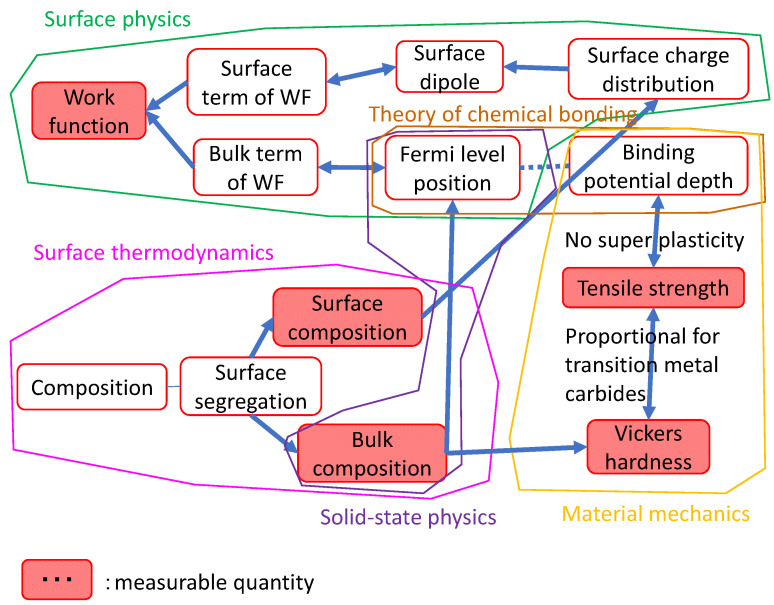
Relationships among factors that contributing to the work function, compiled from descriptions in books and review articles.

The work function is a material property that determines the energy barrier to electron transfer in many devices such as transistors, batteries, and solar cells. Although it is a material property, the value is determined not only by the bulk term (the bulk composition and bulk structure) but also by the surface term (the surface composition, which is not necessarily the same as the bulk composition, and surface atomic arrangement and structures, including the arrangement of steps). Figure 6 shows various material properties that contribute to the work function. In the devices mentioned above, the main functional material is sandwiched between two metallic electrodes, one with low work function and the other with high work function. Most materials with low work function, such as alkali metals, are very reactive. Among low-work-function materials, transition metal carbides (TMCs) and nitrides (TMNs) are less reactive and relatively easy to handle in device processing. Carbides such as TiC and TaC are in practical use.

TMCs are non-stoichiometric compounds, and carbon atoms often deviate from a 1:1 ratio, resulting in the formula TMCx (x < 1). The work function is affected by the stoichiometry, but only two experimental results on the effects for well-defined surfaces have been reported [33]. First-principles calculations of these two systems have also been reported [34]; they show that carbon deficiency does not affect surface term of the work function. In addition, first-principles calculations have shown that the surface term of the work function of other TMCs remains constant under a carbon deficiency. Therefore, the carbon deficiency affects only the bulk term of the work function. Thus, the question is how to estimate the bulk term of the work function. From the origin of the work function [35], the author found that the Vickers hardness can be used as one measure of the bulk term of the work function of TMCs and TMNs in general [36]. Figure 6 was compiled on the basis of the above consideration. When this diagram is created and published, other researchers who are not familiar with the work function but need to control it for their devices can use it as a reference without following the author’s entire thought process as described in [36].

## 3. Relationship between Material Properties

**Figure 7:**
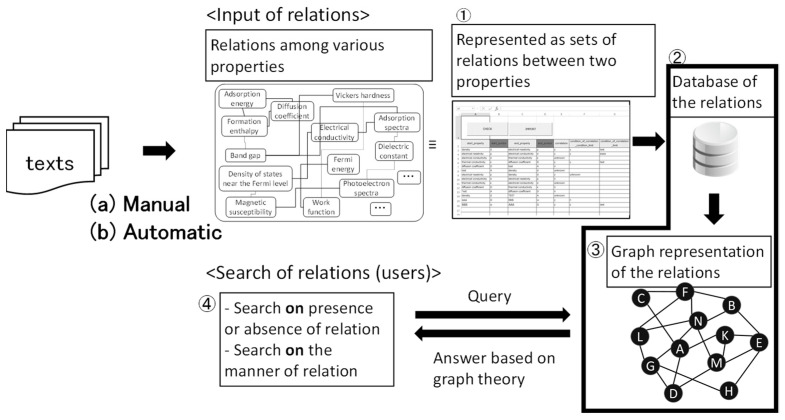
Schematic structure of the proposed system, which enables searches for relationships among material properties.

If a diagram of the relationships between various material properties such as Figure 5 is stored as a database and shared among many material scientists, material development is expected to be greatly accelerated. Consequently, the author proposed a system composed of a database of relationships between various material properties and a search tool for the database [16,17,37,38] as shown schematically in Figure 7. Many relationships on material properties, which are given literally, are extracted as pairs of two material properties from texts either by (a) manually, where a person reads textbooks and learns the relationships like Figure 5 and Figure 6, or by (b) automatically using natural language processing techniques and a computer. Extracted pairs of two material properties are input into a database (<Input of relations> in Figure 7). The database of sets of material property pairs is represented as a graph. Users search relations from the database represented as a graph (<Search of relations (users)> in Figure 7). The characteristic feature of the relationship database is its graph-type (network-type) structure, which consists of nodes (material properties) and edges (relations between material properties). This database is completely different from conventional material databases, which contain material names or compositions and the values of material properties such as melting point, density, and dielectric constant. There are no numerical values in the database. Like a train map, this database describes connections. The contents are not numerical data but words such as density. The sources of scientific principles are mainly literal (including mathematical formula), not numerical. Literal information describes essentially universal relationships independent of specific material compositions. Numerical data are useful for specific material systems.

**Figure 8:**
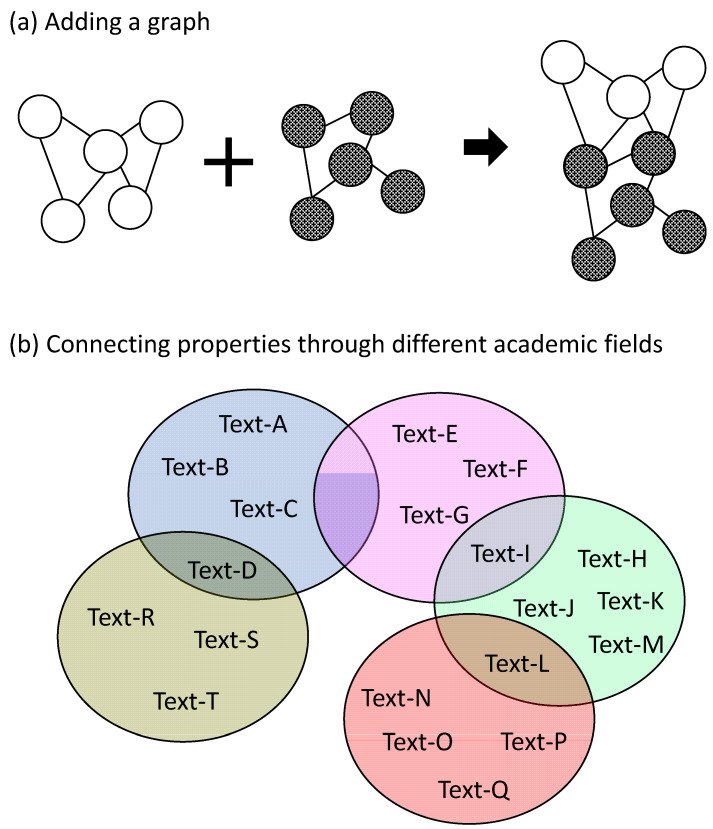
Graph-type database that enables the easy addition of a graph (**a**) and easy expansion of academic fields (**b**), where different colors indicate different academic fields such as materials mechanics, solid-state physics, and chemical thermodynamics.

**Figure 9:**
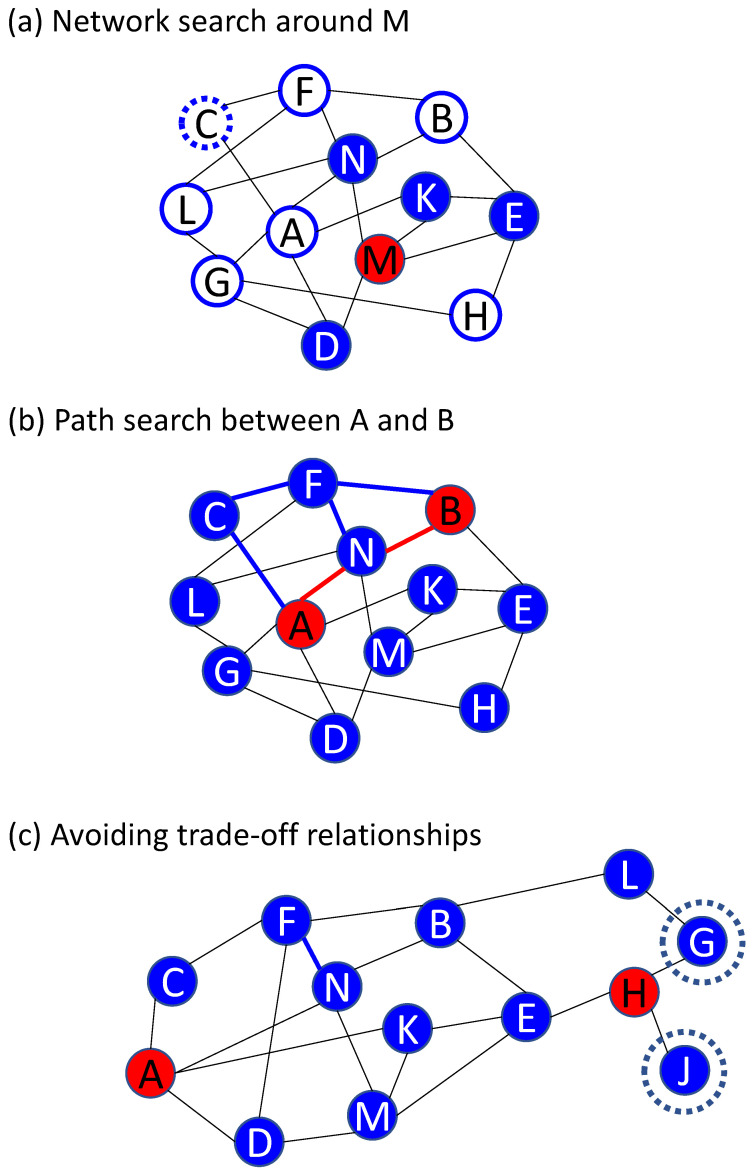
Two basic searches (**a**) network search and (**b**) path search, and search for avoiding trade-offs (**c**), which can be realized by combining path search and network search.

The advantage of a graph-type database is that it is easy to add or subtract data on connections as shown in Figure 8a. Consequently, it is easy to expand the area of scienfitic principles in the relationship database by connecting a material property mentioned in two textbooks in different academic fields (Figure 8b). Basic techniques for searching for relationships (connections) have been established in the framework of graph theory in mathematics [39] and are widely used in society, for example, in route searches of a train map. Graph-type databases are searched mainly by network searches and path searches, as shown in Figure 9. Here, each node (A, B, C, etc.) represents a material property such as density, thermal conductivity, or Vickers hardness, and each edge shows the relationship between two connected properties. Using a network search, one can, for example, find the material properties that affect the target property M. One example in which a path search is useful is when a material modification that increases material property A causes an unexpected decrease in material property B, which is undesirable. By searching the paths from A to B, one can find relationships that might cause the decrease in B with increasing A on these paths. It is also possible to search for possible ways of avoiding trade-off relationships (Figure 9c) by combining a path search and a network search, for example, by finding nodes that do not have a path to A without passing through H (J in Figure 9c) or finding nodes that connect directly to H but have a long path from A (H in Figure 9c). A node with a long path is usually expected to have less effect on a target node (=property), because there are many other nodes that affect the target node, which are used to avoid a trade-off relationship between A and H.

## 4. Computer Systems

**Figure 10:**
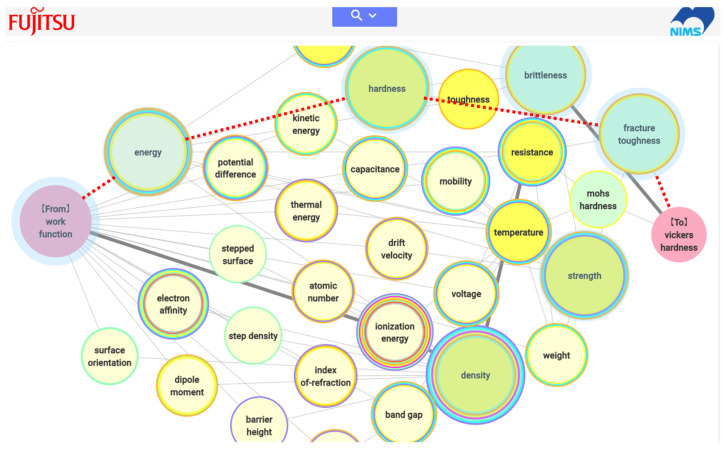
Computer system results screen showing a path search between the material properties of work function and Vickers hardness. Red dotted lines are shown for comparison with the manually compiled relationship in Figure 6.

**Figure 11:**
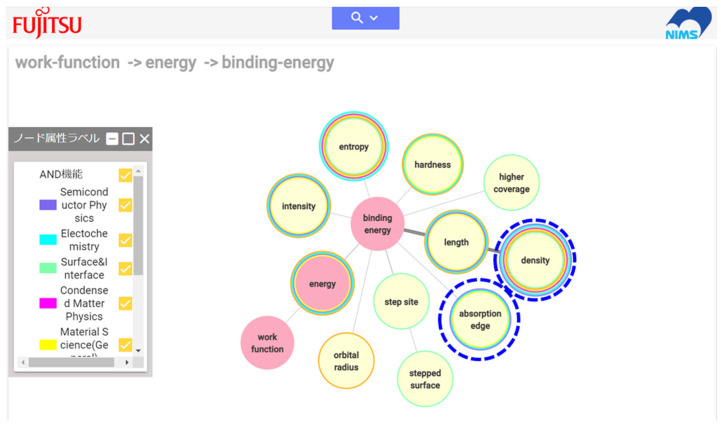
Results screen for sequential network search starting from work function.

Although the small system shown in Figure 7 has been developed and demonstrated [16,17,40], the number of material properties and relationships stored in the system is quite limited because the relationships between material properties were extracted manually. Computer technology for automated relationship extraction is essential for practical use. The author has collaborated with a company to realize automated relationship extraction from several textbooks on materials science, and a prototype system has been developed as a result of this collaborative project [41]. The relationships between material properties automatically extracted from the 12 textbooks listed in Table 1 are currently included in the web-based system. Figure 10 shows an example of the system output for a path search (Figure 9b) between work function and Vickers hardness, whose relationship was explained in Section 2.3. The descriptions in the textbook are not the same as those the author read, but the system also suggests the possibility of estimating work function values from Vickers hardness (there is a connection), and the properties shown in Figure 6 and Figure 10 (path with red dotted lines) show considerable overlap. In the computer system, a path with nodes (material properties) appearing in the largest number of academic fields (represented by the colored circles around the material properties) is shown with thick edges, indicating the most multidisciplinary path. An example of the system output for a network search is shown in Figure 11. Because it is not commonly known that the work function is related to the Vickers hardness, a network search would be useful for finding properties that can be used to estimate the work function. In this case, a network search beginning with a target property (here, the work function) is used.

The search result in Figure 11 uses the trace function (sequential network search, Figure 9a while retaining the previous network search results); the search begins at work function and reaches binding energy. This result suggests that properties such as density and absorption edge might be used in addition to hardness to estimate the work function. For TMCs, it is expected that experimental results on the effect of carbon deficiency on density may exist, but not results on absorption edges. It is reasonable to consider that density is a measure of binding potential depth in Figure 6, because the density would increase if the bonds in the carbides become stronger (that is, the binding potential is deeper) when both molar mass and lattice constant decrease because of carbon deficiency.

**Figure 12:**
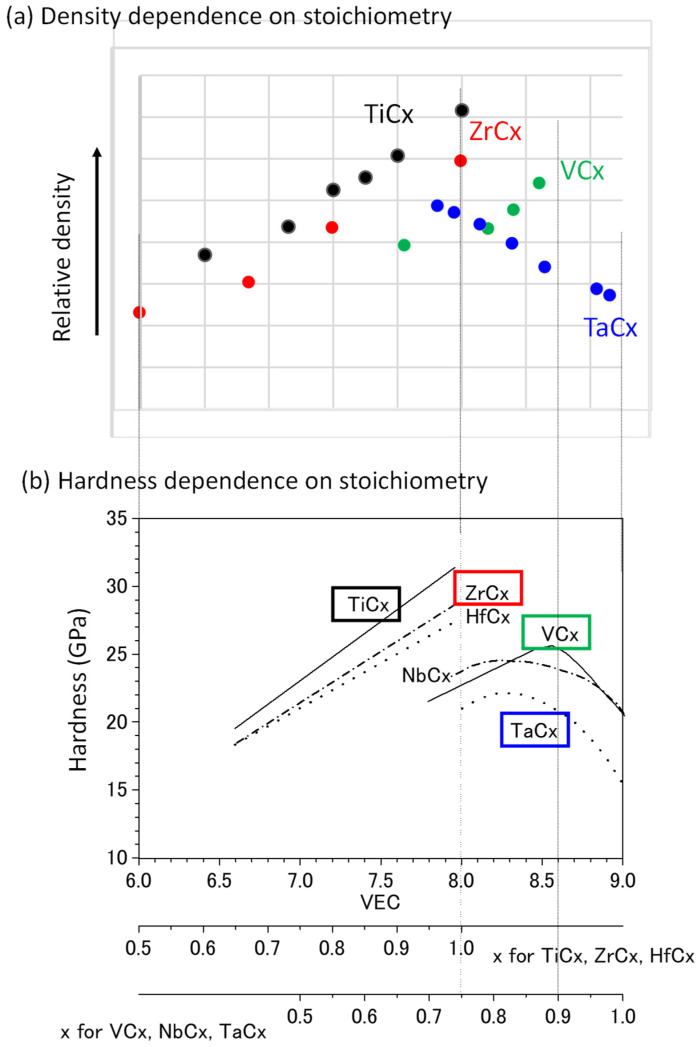
Composition dependence of density of TMCs (**a**) and with that of Vickers hardness [36] (**b**) for comparison. VEC is the abbreviation of “valence electron concentration” [36].

The author checked references on the density of TMCs with carbon deficiency. The effect of carbon deficiency on the density for TiCx [42] and ZrCx [43] (group IV TMCs) and VCx [44] and TaCx [45] (group V TMCs) is shown in Figure 12a, where the density is calculated from lattice constants obtained by X-ray diffraction measurements and the molar mass in the stoichiometry given in the references. In Figure 12b, the effects of carbon deficiency on hardness, which were previously used as a measure of the bulk term of the work function, are also shown for comparison. The absolute values of the density clearly depend on the atomic radius of transition metals. Therefore, the density is plotted as a relative value, and only the qualitative dependence of density on the stoichiometry is considered. For TiCx and ZrCx, whose phase diagrams show a wide region of one carbon-deficient phase, the density decreases monotonously with increasing carbon deficiency (decreasing x), as demonstrated in Figure 12a, in agreement with the trend of hardness in Figure 12b. For VCx and TaCx, the density is expected to increase with increasing carbon deficiency near stoichiometry (0.9 < x <1.0) from hardness change with carbon deficiency. Although TaCx shows the expected dependence on carbon deficiency, density values for 0.9 < x <1.0 are missing for VCx. The density of VCx decreases with carbon deficiency for x < 0.87, which is consistent with the hardness trend. In the phase diagram of the binary system of V and C [46], VCx exists in the range 0.66 < x < 0.89 at 1650 °C, where the concentration of C dissolved in metallic V is the maximum. The above range is in agreement with the data range for the density in Figure 12a. Therefore, it is considered that the density, like the Vickers hardness, is also useful as a measure of the bulk term of the work function for VCx. TaCx exists in the range 0.68 < x < 0.99 at 2843 °C, where the concentration of C dissolved in metallic Ta is the maximum. Because the composition at which the hardness is maximum is somewhat unclear, it is difficult to discuss the behavior of TaCx near the lower limit of x. In summary, it appears that the density can be used as an indicator of the effect of carbon deficiency on the bulk term of the work function in TMCs, at least in the composition range in which the carbon deficiency is smaller and the TMCx phase exists in the phase diagram.

In the above example, the density of carbon-deficient TMCs was checked manually because there is no retrievable database. However, automated data collection and data presentation, as shown in Figure 12b, should be possible in principle, which would assist an individual researcher in the design process illustrated in Figure 1.

The system presented here is still a prototype. The development of a product and commercialization of the product is necessary in future. In addition, many additional functions such as quantitative relationships, arranging tie-ups with numerical database and machine learning are desired. Finding a new relations based on the structure of the graph database could be also explored, because there are considerable numbers of scientific principles represented in a similar form such in particle mechanics and geostatics and electric field and magnetic field in electromagnetics.

## 5. Conclusions

A materials informatics method that uses knowledge of scientific principles as well as numerical data was proposed. The use of systematic knowledge of scientific principles enables a broader perspective that is less limited by commonly used approaches. Some examples of material search and prediction using very little experimental data were shown to demonstrate the advantage of using scientific principles. Then, a system consisting of a database of knowledge on the relationships between material properties and a relationship search function, which is being developed by the author and collaborators, was presented. Finally, the author’s discovery that work function values can be estimated from the density of materials when the effect of carbon deficiency in TMCs is considered is presented to demonstrate the usefulness of the system.

## 6. Patents

In the article, the following five patents, (1) property relationship database and search system, (2) those with options on priority, (3) those with modified search, (4) those with user information including search history, (5) those with combined search used for avoiding trade-offs, for example, are related.

(1) Search System, Search Method, and Physical Property Database Management Device, Japanese Patent #6719748, US Patent allowed (publication # 2019/0139279).
(2) Search System, Search Device, and Search Method, Japanese Patent # 6876344, US Patent # 11163829
(3) Search System, and Search Method, PCT/JP2019/028188.
(4) Search System, and Search Method, PCT/JP2019/030108.
(5) Search System, and Search Method, Japanese Patent publication #2021-012502.

## Figures and Tables

**Table 1:** List of textbooks used for the prototype system.

Book Title	Author(s)	Publisher	Year
Fundamentals of Materials Science	Eric J. Mittemeijer	Springer	2011
Understanding Materials Science	Rolf E. Hummel	Springer	2004
Materials Handbook	François Cardarelli	Springer	2018
The Chemical Bond I–III	D. Michael P. Mingos, ed.	Springer	2016
Ceramic Materials: Science and Engineering	C.Barry Carter, M. Grant Norton	Springer	2013
Electrochemistry for Materials Science	Walfried Plieth	Elsevier	2008
Solid State Electrochemistry I: Fundamentals, Materials and their Applications	Vladislav V. Kharton	WILEY	2009
Electronic Properties of Materials	E Hummel	Springer	2011
Physics of Semiconductor Devices	Simon M. Sze, Kwok K. Ng	WILEY	2006
Principles of Surface Physics	Friedhelm Bechstedt	Springer	2003
Physics of Surfaces and Interfaces	Harald Ibach	Springer	2006
Solid Surface Physics	Heribert Wagner	Springer	1979

## Data Availability

The data presented in this study are available on request from the author.
